# Transactions of the American Association of Obstetricians and Gynecologists

**Published:** 1890-06

**Authors:** 


**Transactions of the American Association of Obstetricians and Gynecologists.** Vol. II. for the year 1889. Standard 8vo, pp. xl.–397. Philadelphia: William J. Dornan, printer. Cloth, $5.00; half-Russia, $6.00.

The second volume of this association's work presents quite as attractive material for examination as did the first, which is paying the book a high compliment.

The following table of contents will furnish the reader with some idea of the value of the work done by the association during its three days' session :

The President's Annual Address, by William Henry Taylor, M. D.; Congenital Sinus of the Urachus, Abdominal Incision. Recovery, with Remarks, by A. Vander Veer, M. D.; A Case of Extra-uterine Pregnancy. Operation, Recovery, by L. S. McMurtry, M. D.; Treatment of the Ruptured Parturient Uterus, with report of two cases, by Charles A. L. Reed, M. D.; two cases of Intra-uterine Amputation of the Forearm, by Joseph Price, M. D.; Rectification of Face Presentations, by Rollin L. Banta, M. D.; Flap-splitting in Perineorrhaphy, with Special Reference to Tait's Operation, by X. O. Werder, M. D.; The Necessity of Recognizing Three Planes in the Obstetric Pelvis, by William Wotkyns Seymour, M. D.; How the Refinements of Abdominal Surgery have Influenced General Surgery, by David Barrow, M. D.;

Some Accidents and Complications Incident and Subsequent to Abdominal and Pelvic Operations, by Joseph Hoffman, M. D.; A Study or the Pathology and Treatment of Intra-pelvic Inflammations, by L. S. McMurtry, M. D.; Vaginal Hysterectomy, by E. E. Montgomery, M. D.; The Dry Extra-peritoneal Treatment of the Stump in Hysterectomy, by Joseph Price, M. D.; Successful Removal of a Fibroma of the Right Ovary during Pregnancy, by J. Henry Carstens, M. D.; Ligatures and Sutures. What Material shall be Used? by Clinton Cushing, M. D.; A Few Considerations on Peritoneal Effusions after Intra-peritoneal Operations, by William H. Meyers, M. D.; Reasons for Drainage in Ovariotomy, by Hampton E. Hill, M. D.; Umbilical Hemorrhage, its Treatment, by Llewellyn Eliot, M. D.; The Management of the Perineum during Labor, by Augustus P. Clarke, M. D.; A Report of Ninety Cases of Rapid Dilation of the Uterine Canal for the Cure of Dysmenorrhea and Sterility, by Franklin Townsend, M. D.; A Case of Metremphysema, by Thomas E. McArdle, M. D.; Some Points in the Diagnosis of Pyosalpinx, by Rufus B. Hall, M. D.; The Forceps as a Means of Rotating the Head in Labor, by Edward J. Ill, M D.; The Need of Specially Trained Men for Abdominal Surgery in Remote Districts, by George R. Dean, M. D.; A Remarkable Case of Nymphomania, and its Cure, by William S. Stewart, M. D.; A Contribution to the History of Ovariotomy on the Insane, by W. P. Manton, M. D.; New Observations Concerning the Functions of the Round Ligaments, by J. H. Kellogg, M. D.; Peculiarities of Abortion from Artificial Causes, by Hamilton A. West, M. D.; The Animal Suture, its Place in Surgery, by Henry O. Marcy, M. D.; Some Neuroses of the Menopause, by William J. Conklin, M. D.; Sapremia and Septicemia during the Puerperal Period, by William S. Gardner, M. D.; The Genu-pectoral or Knee-elbow Position in Obstetrics, by W. H. Wenning, M. D.; Discussion on Craniotomy. I. Is Craniotomy Justifiable on Living Children? by George H. Rohé, M. D.; II. Its Alternatives; Abortion, by William Warren Potter, M. D.; Premature Labor, by Joseph Hoffman, M. D.; Forceps and Version, by James P. Boyd, M. D.; The Cesarean Section, by E. E. Montgomery, M. D.; The Cesarean Section, by A. Vander Veer, M. D.; Laparaelytrotomy, by Charles A. L. Reed, M. D.; The Porro Operation, by Joseph Price, M. D.; III. Conclusions: A Résumé of the Whole Subject, by William H. Wathen, M. D.

The discussions are spirited and elaborate, giving a force and effect to the papers that may well serve as a model for society work.

The book is handsomely printed on tinted paper, and the illustrations, thirty-six in number and two plates, are well executed. Taken all in all it is the most beautifully gotten up volume of society transactions we have yet seen, and it should find a place in the library of every physician who desires to keep pace with such literature.

